# Exploration of the power of routine surveillance data to assess the impacts of industry-led badger culling on bovine tuberculosis incidence in cattle herds

**DOI:** 10.1136/vr.103201

**Published:** 2015-09-15

**Authors:** C. A. Donnelly, A. I. Bento, A. V. Goodchild, S. H. Downs

**Affiliations:** 1 MRC Centre for Outbreak Analysis and Modelling, Department of Infectious Disease Epidemiology, School of Public Health, Faculty of Medicine, Imperial College London, Norfolk Place, London W2 1PG, UK; 2 Department of Epidemiological Sciences, Animal and Plant Health Agency-Weybridge, Woodham Lane, New Haw, Addlestone, KT15 3NB, UK

**Keywords:** Epidemiology, Statistics, Tuberculosis (TB)

## Abstract

In the UK, badgers (*Meles meles*) are a well-known reservoir of infection, and there has been lively debate about whether badger culling should play a role within the British Government's strategy to control and eventually eradicate tuberculosis (TB) in cattle. The key source of information on the potential for badger culling to reduce cattle TB in high-cattle-TB-incidence areas remains the Randomised Badger Culling Trial (RBCT). In late 2013, two pilot areas were subjected to industry-led badger culls. These culls differed importantly from RBCT culling in that free-ranging as well as cage-trapped badgers were shot, and culling took place over a longer time period. Their impacts will be harder to evaluate because culling was not randomised between comparable areas for subsequent comparisons of culling versus no culling. However, the authors present calculations that explore the power of routine surveillance data to assess the impacts of industry-led badger culling on cattle TB incidence. The rollout of industry-led culling as a component of a national cattle TB control policy would be controversial. The best possible estimates of the effects of such culling on confirmed cattle TB incidence should be made available to inform all stakeholders and policy-makers.

## Introduction

Bovine tuberculosis (TB) is a zoonotic disease caused by *Mycobacterium bovis.* Cattle are the main host species. However, there are other domestic and wild reservoirs, the most important of which in the UK is the Eurasian badger (*Meles meles*) (Krebs and others 1997). The control of bovine TB in British cattle is difficult due to the reservoir of infection in badgers and the limitations of the diagnostic tests available to diagnose *M. bovis* infection in cattle.

The key experimental evidence on the potential for badger culling to reduce cattle TB in high-cattle-TB-incidence areas remains the Randomised Badger Culling Trial (RBCT). The RBCT estimated the impacts of two potential badger culling policies on cattle TB incidence by comparing the incidence of cattle TB in 100 km^2^ areas randomised to receive annually either widespread (‘proactive’) culling or localised (‘reactive’) culling or no culling (‘survey-only’) in the vicinity of herds with confirmed TB incidents (Donnelly and others 2003, 2006; Bourne and others 2007). Each of these experimental treatments was assigned to 10 areas in England with areas being grouped into geographically matched sets of three, referred to as triplets. Analyses compared the incidence of herd TB incidents within culled areas with the incidence in matched ‘survey-only’ areas using log-linear Poisson regression models, which corrected for triplet, the log-transformed number of baseline herds at risk and the log-transformed number of confirmed herd TB incidents within the area in a three-year historic period (Donnelly and others 2003, 2006; Bourne and others 2007; Jenkins and others 2008, 2010). An effect was only observed in relation to TB incidents where infection with *M. bovis* was confirmed by postmortem evidence, and the official TB-free status of the herd withdrawn (OTFW). Proactive culling was found to significantly decrease confirmed cattle TB incidence within RBCT areas, but during the period of culling, it was also found to significantly increase confirmed cattle TB incidence in the 2 km outside of proactively culled RBCT areas (Donnelly and others 2006, Bourne and others 2007).

The RBCT also demonstrated that ‘reactive’ culling significantly increased confirmed cattle TB incidence within RBCT areas (Donnelly and others 2003, Vial and Donnelly 2012). It has been argued that this increase in incidence was caused by perturbation of badger social behaviour (Woodroffe and others 2006a), which led to an increase in transmission of the infection both between badgers (Woodroffe and others 2006b) and from badgers to cattle. Thus, the effects of culling depend upon geographical scale of the area culled (Donnelly and others 2007).

In 2011, the British Government announced their intention to license groups of farmers and their agents to cull badgers (at the farmers’ expense) as part of the Government's TB-control strategy. In late 2013, pilot culls were conducted in West Somerset and West Gloucestershire while expressions of interest for badger control licences for additional areas have been sought. The design of the badger culling policy was informed by results from the RBCT, although the culling method and duration differed (Munro and others 2014). In particular, areas were required to be at least 150 km^2^ to increase the benefits of decreased incidence within the culled area relative to the increased incidence of up to 2 km outside the culled area. Culls were required to reduce the estimated badger population by 70 per cent, and were to be repeated annually for at least four years (Defra 2013, 2014; Ares and Pilbeam 2014).

Because the 2013 pilot culls did not achieve their aims in reducing badger populations by at least 70 per cent and missed their welfare target (Munro and others 2014), no new areas were licensed for industry-led culling in 2014 (Ares and Pilbeam 2014). However, further training of contractors has been conducted, and the first follow-up culls have been undertaken in 2014 in the two areas (West Somerset and West Gloucestershire) that received pilot culls in late 2013. The licensing of additional areas will depend on the outcome of the first follow-up culls in the initial two areas.

Although the pilot culls were undertaken ‘in order to confirm the effectiveness and humaneness of controlled shooting’ (paragraph 35), there was also a government commitment to compare cattle TB incidence in culled areas to that in ‘similar unculled areas to identify any changes in trends that might be attributable to badger control’ (paragraph 40) (Defra 2011). Even if additional areas are subjected to industry-led culls, the impacts of the industry-led culling on cattle TB incidence will be harder to evaluate than were the impacts of RBCT culling because the industry-led culling was not randomised among comparable areas that could be subsequently compared with and without culling. Additional biosecurity advice has also been given to farmers within culling areas, but not in the comparison areas (Paterson 2014). However, the authors present illustrative calculations to explore the power of routine surveillance data to assess the impacts of industry-led badger culling on cattle TB incidence.

In addition to any impact of industry-led badger culling on confirmed cattle TB incidence, the number of herd TB incidents in a given area will depend on the number of cattle herds, the per-annum per-herd baseline incidence, the frequency at which herds are tested for TB as well as on the sensitivity of the diagnostic test(s) employed. Although it will not be possible to separate out the effects of culling from the additional biosecurity advice to farmers, based on plausible culling area sizes and incidence the present calculations will inform stakeholder expectations on what results might be expected in future and the likely delay before significant results (if any) might appear.

## Methods

The expected numbers of OTFW (confirmed) herd TB incidents by year and cumulatively were calculated for areas with and without culling assuming that each area contains 200 (or 100) annually tested herds and a baseline incidence of confirmed herd incidents of 0.15 (or 0.10) per herd per annum, based on the estimated time-dependent impact of RBCT proactive culling within culled areas (Table 1, previously published in Jenkins and others 2008).

**TABLE 1:** Estimated effects of proactive culling on the incidence of OTFW herd incidents inside RBCT areas as published by Jenkins and others (2008)

	Estimate (and 95% CI)
1st to 2nd cull	−3.6% (−33.1% to 38.9%)
2nd to 3rd cull	−12.9% (−38.8% to 24.2%)
3rd to 4th cull	−39.6% (−59.3% to −10.3%)
After 4th cull to end of during-trial period	−31.8% (−48.5% to −9.7%)

OTFW, official tuberculosis-free status of the herd withdrawn; RBCT, Randomised Badger Culling Trial

The impact of industry-led culling will be estimated by the comparison of the observed numbers of OTFW herd incidents in culled and unculled areas, over the same time period. The present null hypothesis is that there is no impact of industry-led culling on the incidence of OTFW herd incidents. The data will be analysed to determine whether there is sufficient evidence for this null hypothesis to be rejected. The authors performed a two-sided test of the null hypothesis, so that the alternative hypothesis would include both a decrease and an increase in cattle TB incidence.

In conducting a hypothesis test, there are two types of possible error:

1. Alpha (α) is used to denote the probability of a *Type 1 Error*, that is the probability of rejecting the null hypothesis when it is actually true (ie, if there is no effect).
2. Beta (β) is used to denote the probability of a *Type 2 Error*, that is the probability of not rejecting (accepting) the null hypothesis when it is actually false (ie, if there is an effect).

*Power* is defined to be (1−β), which is the probability of rejecting the null hypothesis when the null hypothesis is false.

The formula for the required sample size (for each group) to compare two population means (μ_A_ and μ_B_) with a common variance σ^2^ is1
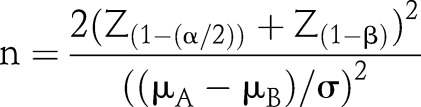
for normally distributed data and a two-sided test.

For the traditional assumption regarding statistical significance, α=0.05 and thus Z_(1−α/2)_=1.960. For 80 per cent power, Z_(1−β)_=0.842, whereas for 90 per cent power, Z_(1−β)_=1.282. Any rounding of n has been to higher values to ensure that each sample size calculation achieves at least the intended statistical power.

A simple sample size calculation can be undertaken using the relationship between the Poisson and normal (Gaussian) distributions such that if Y is Poisson distributed with mean (and variance) μ, then the square root of Y is approximately normally distributed with mean square root of μ and variance 0.25. Thus, denoting the expected numbers of OTFW herd incidents in comparison (CO) and culling (CU) areas Y_CO_ and Y_CU_, respectively, the sample size formula becomes:2
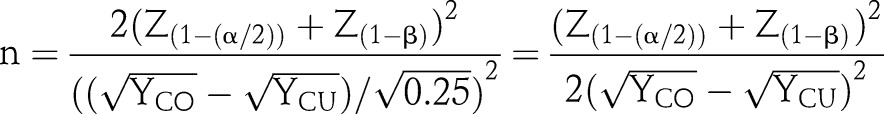


On this simple basis, illustrative sample sizes were calculated corresponding to at least 80 and at least 90 per cent power, assuming the number of herds in the culled and unculled areas were equal and that these herds were at the same baseline risk. Alternatively, equations 1 and 2 can also be used to calculate the power associated with particular numbers of herds under study in culled and unculled areas.

In addition to these calculations, Poisson simulations were used to estimate the power of likelihood ratio tests for differences between culling and comparison areas relating to different numbers of comparison areas per culling area. Illustrative power estimates were obtained from 10 000 simulations per scenario. Follow-up varied from two to four years with 1–4 comparison areas per culling area. The power based on the equation 2 (with one comparison area) was also given for comparison. Each area was assumed to contain 200 (or 100) annually tested herds and to have a baseline incidence of OTFW herd incidents of 0.15 (or 0.10) per herd per annum. The underlying impacts of the industry-led culling were assumed to be the estimates presented in Table 1 (originally published by Jenkins and others 2008).

## Results

Based on the estimates presented in Table 1 (originally published by Jenkins and others 2008), the expected numbers of OTFW herd incidents by year and cumulatively were calculated for areas with 200 annually tested herds (and for areas with 100 annually tested) assuming a baseline incidence of OTFW herd incidents of 0.15 per herd per annum (and assuming a baseline incidence of OTFW herd incidents of 0.10 per herd per annum). Thus, in the absence of badger culling and with no annual trend in incidence, such an area would experience, on average, 30 OTFW herd incidents per annum or 120 OTFW herd incidents over four years (see Table 2).

**TABLE 2:** The expected number of OTFW herd incidents by year and cumulatively within areas with 200 annually tested herds, baseline incidence of confirmed herd TB incidents of 0.15 per herd per annum and impacts of culling as the estimates presented in Table 1

	Expected confirmed herd TB incidents by year			Cumulative expected confirmed herd TB incidents	
	Comparison area (Y_CO_)	Culling area (Y_CU_)		Comparison area (Y_CO_)	Culling area (Y_CU_)
Year 1	30	28.9	Year 1	30	28.9
Year 2	30	26.1	Years 1–2	60	55.0
Year 3	30	18.2	Years 1–3	90	73.2
Year 4	30	20.5	Years 1–4	120	93.8

CO, comparison; CU, culling; OTFW, official TB-free status of the herd withdrawn; TB, tuberculosis

The present calculations—based on the Poisson distribution and the impacts estimated from proactive culling in the RBCT (Jenkins and others 2008)—indicate that, unless very large numbers of culling and comparison areas were under study, one-to-one-matched culling and comparison areas will likely need to be observed for at least three years after culling begins before any significant differences in the incidence of OTFW herd incidents are observed (Table 3). One-to-one-matched pairs of culling and comparison areas with lower baseline incidence of OTFW herd incidents require longer follow-up to achieve the same level of statistical power (Table 3).

**TABLE 3:** Illustrative sample sizes in terms of the number of matched pairs of culling and comparison areas required for at least 80 and at least 90 per cent power

	200 annually tested herds per area and baseline incidence of OTFW herd incidents of 0.15 per herd per annum (*or 0.10 per herd per annum*)		100 annually tested herds per area and baseline incidence of OTFW herd incidents of 0.15 per herd per annum (*or 0.10 per herd per annum*)	
Time under observation	At least 80% power	At least 90% power	At least 80% power	At least 90% power
1 year	397 (*595*)	531 (*797*)	793 (*1190*)	1062 (*1593*)
2 years	37 (*56*)	50 (*74*)	74 (*111*)	99 (*148*)
3 years	5 (*7*)	7 (*10*)	10 (*14*)	13 (*19*)
4 years	3 (*4*)	4 (*5*)	5 (*8*)	7 (*10*)

OTFW, official tuberculosis-free status of the herd withdrawn

The simulation results in Table 4 demonstrate that although multiple comparison areas per culling area increases the power, two years of follow-up of up to six culling areas does not provide a suitable level of statistical power.

**TABLE 4:** Illustrative power estimates were obtained from 10 000 simulations per scenario. Follow-up varied from two to four years with 1–3 comparison areas per culling area

Number of culling areas	Number of comparison areas per culling area			
	1 per culling area (Equation) (%)	1 per culling area (Simulation) (%)	2 per culling area (Simulation) (%)	3 per culling area (Simulation) (%)
(a) Based on 2 years of follow-up				
2	9.4	9.8	11.7	12.5
3	12.1	12.7	15.3	16.6
4	14.8	15.1	18.3	20.2
5	17.4	17.6	21.9	24.1
6	20.0	19.7	25.4	27.6
(b) Based on 3 years of follow-up				
2	46.1	45.4	56.5	61.6
3	62.6	61.9	73.9	79.2
4	75.0	74.7	85.8	89.7
5	83.8	83.9	92.8	95.2
6	89.7	89.7	96.2	97.4
(c) Based on 4 years of follow-up				
2	72.6	73.2	84.6	88.6
3	88.0	88.8	95.3	97.2
4	95.2	95.5	98.7	99.4
5	98.2	98.3	99.8	99.9
6	99.3	99.4	100.0	100.0

The power based on the equation given above (with one comparison area) was also given for comparison. Each area was assumed to contain 200 annually tested herds and to have a baseline incidence of OTFW herd incidents of 0.15 per herd per annum. The impacts of culling were assumed to be the estimates presented in Table 1

OTFW, official tuberculosis-free status of the herd withdrawn

## Discussion

The present calculations—based on the Poisson distribution and the impacts estimated from proactive culling in the RBCT (Jenkins and others 2008)—indicate that culling and comparison areas will likely need to be observed for at least three years after culling begins before any significant differences in the incidence of OTFW herd incidents are observed. Culling and comparison areas with lower baseline incidence of OTFW herd incidents require longer follow-up to achieve the same level of statistical power.

The calculations presented in this study have not allowed for between-area variation beyond that expected due to chance (ie, Poisson variation). It is clear from equation 2 that extra-Poisson variation (ie, variance beyond that expected by chance) in the counts of OTFW herd incidents will increase the required sample sizes with n being proportional to σ^2^, the variance of the square roots of the observed counts. Thus, for example, twice the expected variation (in the square rooted counts) will double the required sample sizes. Such extra-Poisson variation would occur if herd incidents tend to occur in groups rather than as isolated events. Extra-Poisson variation was observed within the RBCT (Jenkins and others 2008). Therefore, the results presented here should be viewed as maximum estimates for power of comparisons between culling and comparison areas for a given sample size. In reality, comparison areas will be carefully matched to culling areas using geographic information system analysis (Brunton and others 2015).

It is clear in Table 4 that although multiple comparison areas per culling area increases power, two years of follow-up does not provide a suitable level of statistical power for as many as six areas. Although the selection of multiple comparison areas is important because it will increase precision and guard against comparison areas being lost completely due to subsequent conversion into culling areas, their inclusion is unlikely to substantially shorten the duration of the follow-up. This loss of comparison areas may be particularly challenging for the analysis if the probability of a comparison area subsequently being culled increases with the incidence of OTFW herd incidents in the area, causing the mean incidence in comparison areas that remained unculled to be lower than average.

Two pilot culls took place in 2013. If, for example, five culling areas were to begin culling in 2015, and five more were to begin culling in 2016, and the culling had a similar effect to that of proactive culling as implemented in the RBCT then it is likely that significant differences in the incidence of OTFW herd incidents between culling and comparison areas could be observed in 2020 (when 12 areas had been observed for four or more years). It is possible that significant differences could be observed in 2019 (when seven areas had been observed for four or more years and five had been observed for only three years).

The licence criteria set out by Natural England relating to the proportion of badgers to be removed by culling (Natural England 2014) were based on the results of rigorously conducted RBCT (Donnelly and others 2003; Woodroffe and others 2006a; Jenkins and others 2008, 2010). The industry-led culling is being conducted outside the rigorous requirements of an experimental trial. If the industry-led culling removes a substantially smaller or substantially greater proportion of the badger population than RBCT proactive culling did, the present estimates of sample sizes and years of follow-up required will not be appropriate. Furthermore, the introduction of new control policies in the culling areas that are not introduced in comparison areas means that it will not be possible to distinguish the independent effects of culling, although the effect of culling combined with other policies can still be evaluated.

Beyond the calculations performed here, there is an argument based on non-parametric pairwise comparisons that there should be at the least six culling areas paired with six comparison areas. This lower bound for the sample size is regardless of the impacts of culling on cattle herd incidence. To understand this, consider a study of five culling-comparison-area pairs. An extreme outcome is that all five pairs have higher TB incidence in the comparison area than in the matched culling area. The two-sided P value for this outcome is 2 x (0.5)^5^=0.0625 (ie, greater than the traditional 0.05 threshold for significance); so, it is not possible to obtain a significant two-sided test result with only five culling-comparison-area pairs. Alternatively, consider a study of six culling-comparison-area pairs. An extreme outcome is that all six pairs have higher TB incidence in the comparison area than in the matched culling area. The two-sided P value for this outcome is 2 x (0.5)^6^=0.03125 (ie, less than the traditional 0.05 threshold for significance); so, it is, thus, possible to obtain a significant result with as few as six culling-comparison-area pairs if all the pairwise differences are in the same direction. For studies with six or more matched culling-comparison-area pairs, the power would depend on the underlying probability of the culling area having more (or fewer) OTFW herd incidents than the matched culling area. However, for five or fewer culling-comparison-area pairs, the power is 0 per cent. Therefore, a minimum of six matched pairs of culling and comparison areas are required to use non-parametric methods to detect an effect of the badger culling policy on the incidence of OTFW herd incidents.

Significantly decreased confirmed cattle TB incidence within proactively culled areas was not the only observed effect of proactive culling within the RBCT (Donnelly and others 2006; Jenkins and others 2008, 2010). There was also significantly increased confirmed cattle TB incidence up to 2 km outside the proactively culled areas (Donnelly and others 2003). Thus, cattle TB incidence will be monitored up to 2 km outside of industry-led culling areas, compared with cattle herds on land up to 2 km outside of comparison areas. The equations presented here could be used to perform similar calculations for the power of a study to detect any increased risks on land outside of those areas subjected to industry-led culling.

The rollout of industry-led culling as a routine component of a national cattle TB control policy would be controversial. However, in order to inform any such debate, it is crucial that analyses are undertaken to provide the best possible estimates of the effects of such culling on cattle TB incidence to inform all stakeholders and policy-makers.
